# Supplementary material for: A Jacob/nsmf gene knockout does not protect against acute hypoxia- and NMDA-induced excitotoxic cell death
Source: Mol Brain. 2023 Feb 11;16:23. doi: 10.1186/s13041-023-01012-2 (PMC9921040; doi:10.1186/s13041-023-01012-2)
Supplement: Supplementary file 1 — Additional file 1. Extended materials and methods, detailed information on statistics. [file 13041_2023_1012_MOESM1_ESM.docx]

**Additional File 1**

**Extended materials and methods, detailed information of statistics**

**Animals**

Male C57BL/6J mice and Jacob/nsmf knockout mice^1^ were bred and maintained in the animal facility of the Leibniz Institute for Neurobiology, Magdeburg, Germany. Characterization of nsmf-/- mice and the lack of Jacob expression in those mice can be found in Spilker et al.^1^. Animals were housed in groups of up to 5 in individually ventilated cages (IVCs; Green line system, Tecniplast) under controlled environmental conditions (22 °C +/- 2 °C, 55 % +/- 10 % humidity, 12 h light-dark cycle, lights on at 06:00 am). Food and water were provided *ad libitum*.

**Culturing of murine hippocampal slices (OHSC), stimulation**

OHSC were prepared according to a previously published protocol^2^. Slices were prepared from P7-P9 Jacob/Nsmf knockout mice and wild-type littermates from heterozygous breeding and cultured for approximately 11 day. OHSC culturing medium consisted of 50% minimal essential medium (Gibco), 25% heat inactivated horse serum (Gibco), 25mM glucose, 2mM glutamine, 25 mM HEPES, 1x B27 and 1x pen/strep, buffered in HBSS+/+ (containing bivalent cations). For experiments with MK-801 slices were prepared from P12 C57BL6/J mice. Before cell death experiments, the viability of slices was assessed by adding 2 μM propidium iodide (PI) for several hours directly into the culture medium and only slices without (or very little, <10 cells) PI fluorescence were used for subsequent experiments.

For OGD-induced cell death experiments, membranes with OHSC were transferred to 1 ml OGD medium (Ringer solution [124 mM NaCl, 4.83 mM KCl, 1.3 MgSO_4_, 1.97 mM CaCl_2_, 1.21 mM KH_2_PO_4_, 25.6 mM NaHCO_3_ pH 7.4] with 10 mM mannitol or 10 mM glucose as a control) with 3 μM PI. Next, cultures were exposed to 10 min of 95 % N_2_/5% CO_2_ gas flow in a hypoxic chamber (Billupsand, Rothenberg) and kept in the reduced O_2_ conditions for 30 min at 35 °C. Afterwards, the membranes were moved back to their previous medium containing 3 μM PI and imaged at the indicated time points.

For NMDA-induced cell death experiments, OHSC were transferred to the medium containing 50 μM NMDA (Sigma-Aldrich) and 3 μM PI for 30 min. Afterwards, membranes were moved back to their previous medium containing 3 μM PI and imaged at the time points indicated in the figure legend. In order to assure that the observed cell death was NMDAR-dependent, slices were pre- incubated with 10 μM MK-801 (Sigma-Aldrich) for 1 h and during the 200 µM NMDA exposure.

To assess the cell death, the images of PI fluorescence, as well as brightfield images, were acquired on a Zeiss Axioskop 2 with the 4x objective using Nikon NIS software. Analysis was performed in Fiji^3^. The CA1, C3, and DG regions of interest (ROIs) were defined manually based on the brightfield image. Mean pixel intensity was measured in grey-scale images of the PI channel, and individually baseline-corrected for time point 0 (directly after the experiment). The analysis was done by an experimenter blind to genotype and treatment group. For representative images, PI images of different time points were aligned using the Fiji plugin linear stack alignment with SIFT and default settings in the rigid mode. Represented images are contrast-enhanced (equally for all groups).

**Data Analysis**

The results are presented as mean +/- SEM. Data were analyzed using two-way analysis of variance (ANOVA) followed by Bonferroni post-hoc test, and a mixed-effect model analysis. Statistical significance was considered as p<0.05. Data was analyzed and plotted using GraphPad Prism v9.0.2 (Graph Pad Software, San Diego, USA).

**References**

1. Spilker C, Nullmeier S, Grochowska KM, Anne Schumacher A, Butnaru I, Macharadze T, Gomes GM, Yuanxiang P, Bayraktar G, Rodenstein C, Geiseler C, Kolodjziej A, Lopez-Rojas J, Montag D, Angenstein F, Bär J, D’Hanis W, Roskoden T, Mikhaylova M, Budinger E, Ohl FW, Stork O, Zenclussen AC, Karpova A, Schwegler H, Kreutz MR. A Jacob/nsmf gene knockout results in hippocampal dysplasia and impaired BDNF signaling in dendritogenesis. PLoS Genet. 2016;15;12(3):1005907.
2. Grochowska KM, Yuanxiang P, Bär J, Raman R, Brugal G, Sahu G, Schweizer M, Bikbaev A, Schilling S, Demuth HU, Kreutz MR. Posttranslational modification impact on the mechanism by which amyloid-β induces synaptic dysfunction. EMBO Rep. 2017 Jun;18(6):962-981.
3. Schindelin J, Arganda-Carreras I, Frise E, Kaynig V, Longair M, Pietzsch T, Preibisch S, Rueden C, Saalfeld S, Schmid B, Tinevez JY, White DJ, Hartenstein V, Eliceiri K, Tomancak P, Cardona A. Fiji: an open-source platform for biological-image analysis. Nat Methods. 2012 26;9(7):676-82.
